# A meta-analysis comparing hand-assisted laparoscopic right hemicolectomy and open right hemicolectomy for right-sided colon cancer

**DOI:** 10.1186/s12957-020-01869-w

**Published:** 2020-05-07

**Authors:** Mohamed Ali Chaouch, Mohamed Wejih Dougaz, Meriem Mesbehi, Hichem Jerraya, Ramzi Nouira, Jim S. Khan, Chadli Dziri

**Affiliations:** 1 Department B of Surgery, Charles Nicolle Hospital, University Tunis El Manar, Tunis, Tunisia; 2 Department of Colorectal Surgery, Queen Alexandra Hospital, Southwick Hill road, Cosham, Portsmouth, UK

**Keywords:** Right colonic cancer, Right hemicolectomy, Hand-assisted laparoscopy, HALS, Open surgery, Colectomy, Colon neoplasms, Outcomes

## Abstract

**Background:**

Mini-invasive colorectal cancer surgery was adopted widely in recent years. This meta-analysis aimed to compare hand-assisted laparoscopic surgery (HALS) with open right hemicolectomy (OS) for malignant disease.

**Methods:**

PRISMA guidelines with random effects model were adopted using Review Manager Version 5.3 for pooled estimates.

**Results:**

Seven studies that involved 506 patients were included. Compared to OS, HALS improved results in terms of blood loss (MD = 53.67, 95% CI 10.67 to 96.67, *p* = 0.01), time to first flatus (MD = 21.11, 95% CI 14.99 to 27.23, *p* < 0.00001), postoperative pain score, and overall hospital stay (MD = 3.47, 95% CI 2.12 to 4.82, *p* < 0.00001). There was no difference as concerns post-operative mortality, morbidity (OR = 1.55, 95% CI 0.89 to 2.7, *p* = 0.12), wound infection (OR = 1.69, 95% CI 0.60 to 4.76, *p* = 0.32), operative time (MD = − 16.10, 95% CI [− 36.57 to 4.36], *p* = 0.12), harvested lymph nodes (MD = 0.59, 95% CI − 0.18 to 1.36, *p* = 0.13), and recurrence (OR = 0.97, 95% CI 0.30 to 3.15, *p* = 0.96).

**Conclusions:**

HALS is an efficient alternative to OS in right colectomy which combines the advantages of OS with the mini-invasive surgery.

## Introduction

A considerable progress of laparoscopic approach was observed in the management of colon cancer [1, 2]. This approach enhances the postoperative recovery with similar oncological outcomes [3]. On the other side, it is perceived that laparoscopic right hemicolectomy remains more challenging than open right hemicolectomy (OS) with a longer learning curve [4]. This factor limits the mini-invasive right colectomy widespread use [3, 5]. At the beginning of laparoscopic careers, hand-assisted laparoscopic surgery (HALS) may present a safe step to overcome the conventional OS [5]. The surgeon inserts a hand inside the abdomen through a special hand port to facilitate dissection without unsetting the pneumoperitoneum [5]. HALS permits the tactile feedback and proprioception to perform a blunt dissection, a rapid control of unexpected bleeding episodes, and specimen handling and removal and cut down the institutional costs [6]. HALS is surely an easier procedure for laparoscopic surgery beginners, but it must be at least equal to or better than OS in terms of postoperative and oncological results. In addition, benefits of mini-invasive approach after an incision of 5 to 7 cm remain established. Many studies with high level of evidence had assessed the advantages and disadvantages of HALS and OS in colorectal surgery, but these studies included benign and malignant disease [7]. These studies also at the same time analysed right colon, left colon, and rectal neoplasms. Right colonic cancer differs from left-sided cancers in anatomical, genetic, clinical, oncological, prognostic, and survival features [3, 8]. This meta-analysis aimed to compare hand-assisted laparoscopic surgery with open right hemicolectomy for malignant disease.

## Methods

According to PRISMA guidelines, we conducted this meta-analysis. Bibliographic research on January 15, 2020, was undertaken in the following sources: the Cochrane database, PubMed/Embase, and Google scholar. The keywords used were “hand-assisted”, “open surgery”, “conventional open surgery”, “cancer”, “right colon”, “ascending colon”, “transverse colon”, “surgery”, “mini-invasive”, “HALS”, “open”, “colectomy”, and “resection”. We considered randomized clinical trials (RCTs) and controlled clinical trials (CCTs) comparing HALS to OS. No language restrictions and humans were entered. The references list of identified articles was also checked to identify further studies. Patients with right-sided colon cancer (right or transverse colon cancer) undergoing right hemicolectomy as conventional or complete mesocolon excision (CME) were considered for inclusion. Patients undergoing right hemicolectomy for benign lesions were excluded. The methodology evaluation of the studies was evaluated by two authors (MAC and MM). In case of discordance, a discussion with MWD was elaborated. CCTs and RCTs were assessed according to the methodological index of non-randomized studies (MINORS) [9] and CONSORT statement [10], respectively. The outcomes evaluated were overall mortality and morbidity (rates of post-operative 30-day complications), conversion rate, placement of the hand port, blood loss, operative time, time to first flatus, number of harvested lymph nodes, postoperative pain score, wound infection, hospital stay, and recurrence. The following variables were extracted from the retained studies by two authors (MAC and MM): country of origin, study period, study design, gender, BMI, follow-up, number of patients, conversion cases, overall mortality and morbidity (rates of 30-day post-operative surgical and medical complications), conversion rate, operative time (skin to skin operative duration), blood loss, time to first flatus, wound infection, harvested lymph nodes number, incision length, postoperative pain score, hospital stay, and recurrence.

Data from eligible studies were pooled using the RevMan 5.3.5 statistical package and random effects model. For continuous data, weighted mean difference (MD) was measured as an effective measure with 95% confidence intervals (95% CI). For dichotomous variables, odd ratios (OR) were measured with 95% confidence intervals (95% CI). We used the Cochrane *χ*^2^ test (*Q* test) to assess heterogeneity, and we calculated the variance Tau^2^, between studies and the *I*^2^.

## Results

### Studies included

**Fig. 1:**
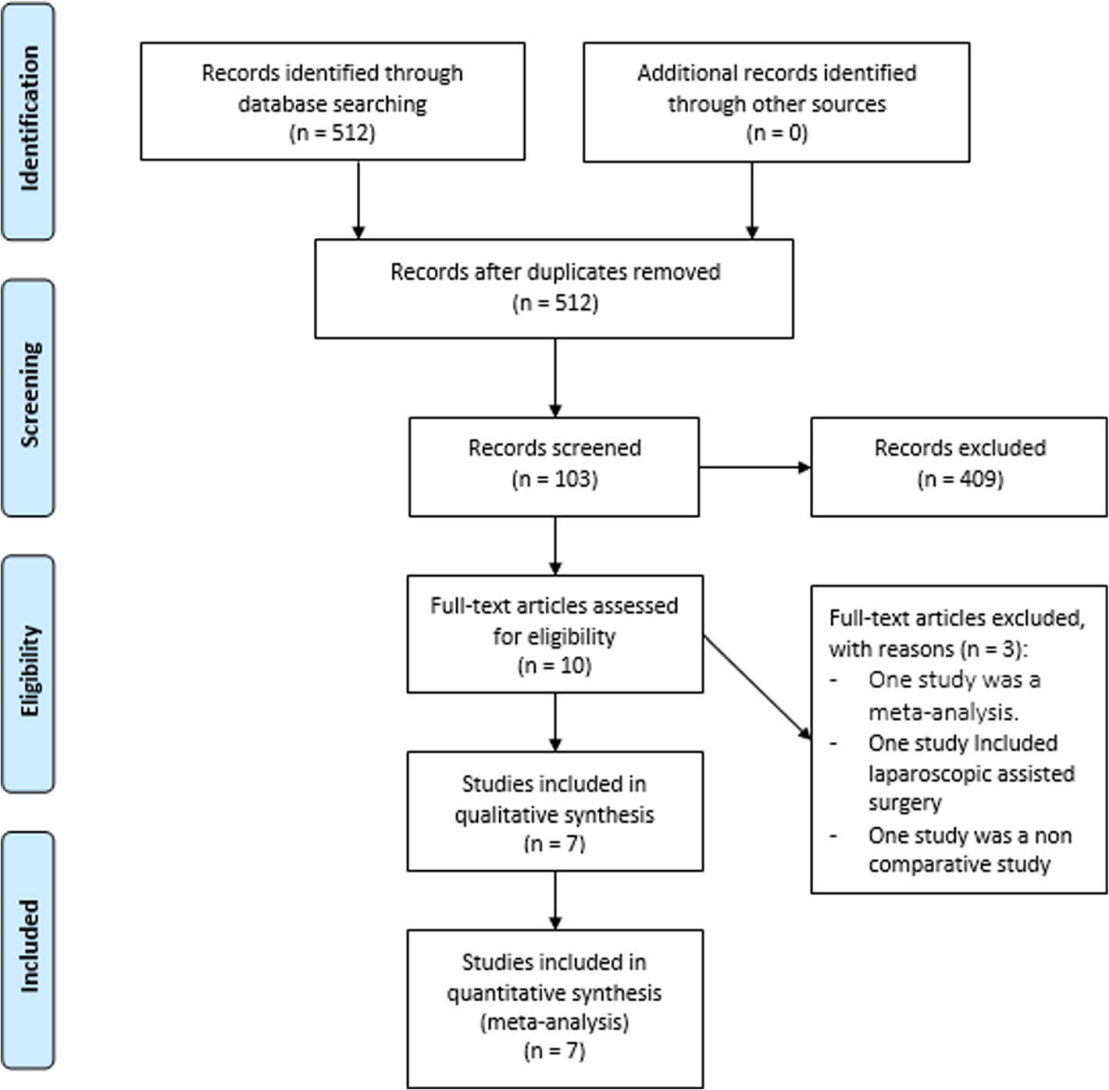
Flow chart of included studies

Seven studies [6, 11–16] published between 2005 and 2018 met the eligibility criteria (Fig. 1). There were two RCTs [6, 14], one prospective comparative non-randomized study [15], and four retrospective and comparative studies [11–13, 16]. They involved 506 patients who underwent HALS (*n* = 238) or OS (*n* = 268). Six studies were from China [6, 11–14, 16], and one study was from South Korea [15]. One study was published in Chinese [12], and all the others were in English [6, 11, 13–16]. Details of patient demographics and studies’ quality assessment for each individual study were summarized in Table 1.

**Table 1:** Characteristics of included studies

First author	Country of origin	Year of publication	Study period	Study design	Type of disease	Number of patients (HALS/OS)	Gender (M:F) HALS/OS	BMI (mean, kg/m^**2**^) HALS/OS	Hand port placement	Tumour staging (HALS/OS)		Follow-up (months) HALS/OS	Conversion cases	Quality assessment	
														MINORS	CONSORT
Sheng 2012 [6]	China	2012	August 2009 to December 2010	RCT, single centre	Right colon cancer	116 (59/57)	32:27/35:22	21.7/22.2	6 cm mid line incision around the umbilicus	**AJCC** I II III IV	7/11 25/24 27/22 0/0	13.3	0	–	16/25
Chung [17]	China	2007	June 2001 to May 2006	RCT, single centre	Right colon cancer	81 (41/40)	25:16/26:14	22/22.8	6.5 cm infra-umbilical midline incision	**DUKES** A B C D	4/5 21/16 15/18 1/1	30/28	3 (7.3%)	–	18/25
Li [16]	China	2015	January 2011 to June 2013	Retrospective, single centre	Obstructive right colon cancer	35 (10/25)	6:4/14:11	22.2/22.3	5.5 cm mid line incision around the umbilicus	**TNM** I II III IV	0/0 2/9 8/16 0/0	NR	0	21/24	–
Wei [11]	China	2018	June 2009 to December 2014	Retrospective, single centre	Right colon cancer	45 (19/26)	9:10/18:8	22.9/21.4	5–7 cm mid line incision around the umbilicus	**TNM** I II III	1/1 12/15 6/10	60/60	NR	19/24	–
Sim [15]	Korea	2013	January 2009 to September 2010	PNR, single centre	Right colon cancer	49 (16/33)	9:7/18:15	22.9/23.8	6–7 cm mid line incision around the umbilicus	**TNM** I II III	9/8 3/14 4/9	NR	0	20/24	–
Sheng [13]	China	2017	May 2012 to April 2014	Retrospective, single centre	Right colon cancer*	150 (78/72)	43:35/40:32	21.7/21.7	6 cm mid line incision around the umbilicus	**TNM** I II III	9/11 35/30 34/31	19.8/20	0	19/24	–
Chi P [12]	China	2005	November 2001 to September 2004	Retrospective, single centre	Right colon cancer	30 (15/15)	6:9/8:7	NR	NR	NR		NR	0	18/24	–

*HALS* hand-assisted laparoscopic surgery, *OS* open surgery, *RCT* randomized clinical trials, *PNR* prospective non-randomized, *NR* not reported

*Complete mesocolon excision was performed

### Outcomes

#### Mortality

Of the seven cohort studies, four studies [12, 14–16] reported the post-operative mortality rate. One patient out of total of 506 patients died in hospital. The patient who died was in the HALS group. He was a 75-year-old man. He presented a myocardial infarction on the 3rd postoperative day [17]. The overall mortality was 0.1% in this review and 0.4% in the HALS group.

#### Morbidity

**Fig. 2:**
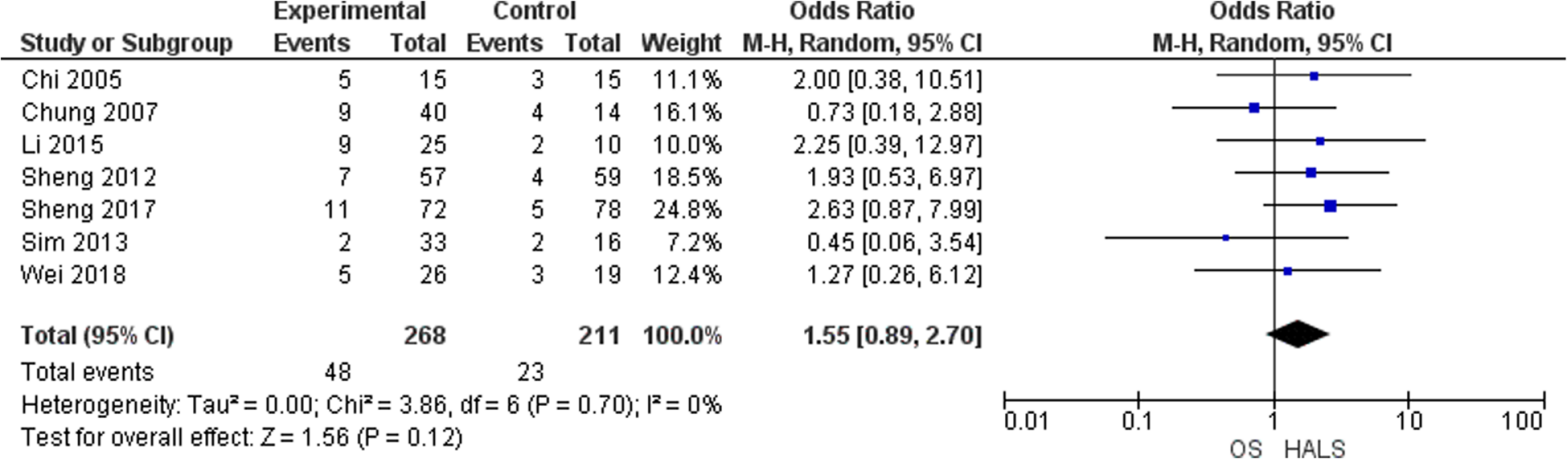
Forest plot of morbidity

Postoperative complications including wound infection [6, 11, 13, 15–17], wound dehiscence [11], intra-abdominal abscesses [6, 11, 13, 17], pneumonia and chest infection [6, 11–13, 16, 17], anastomotic leak or bleeding [6, 11, 13, 16, 17], chylous leakage [13], gastrointestinal dysfunction [6, 11–13, 15], urinary tract infection [12, 16], cardiac event [17], and mental disturbance [11] were collected and analysed. All the included studies [6, 11–16] reported the morbidity rate (Fig. 2) with no difference in terms of morbidity (OR = 1.55, 95% CI 0.89 to 2.7, *p* = 0.12).

#### Blood loss

**Fig. 3:**
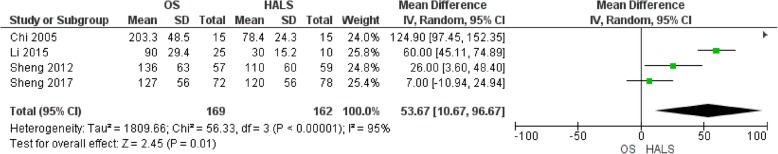
Forest plot of blood loss

This criterion was reported in four studies [6, 12, 13, 16] (Fig. 3). There was significantly less blood loss with HALS than OS (MD = 53.67, 95% CI 10.67 to 96.67, *p* = 0.01). There was a high heterogeneity, Tau^2^ = 1809.663 (*I*^2^ = 95%). Sim et al. [15] has compared the difference between preoperative and post-operative haemoglobin level. It was 1.6 ± 1.1 g/dl in the OS group and 1.3 ± 0.8 g/dl in the HALS group.

#### Conversion rate

Six studies provided details of conversion rate [6, 12–16]. The conversion rate in the HALS was between 0 and 7.3%. One study [17] showed three cases of conversion. In other words, the overall conversion rate was 1.26% in this review. Conversion was related to dense adhesions in two cases and iatrogenic right ureteral transection in one case.

#### Operative time

**Fig. 4:**
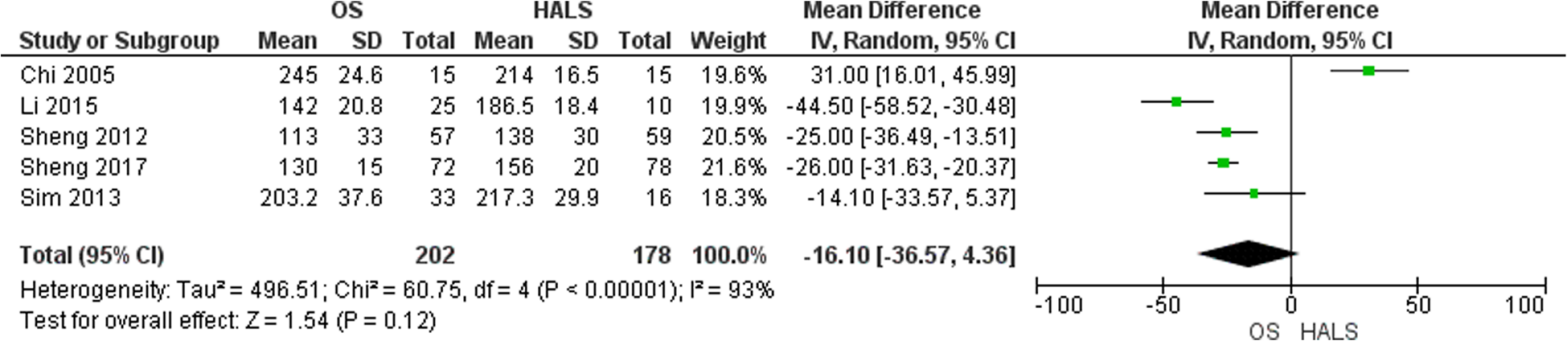
Forest plot of operative time

Five studies reported the operative time [6, 12, 13, 15, 16] (Fig. 4). There was no difference in terms of operative time (MD = − 16.10, 95% CI [− 36.57 to 4.36], *p* = 0.12) with a high heterogeneity rate between the studies, Tau^2^ = 496.51 (*I*^2^ = 93%). In this review, one study [12] reported a shorter operative time in the HALS group.

#### Time to first flatus

**Fig. 5:**
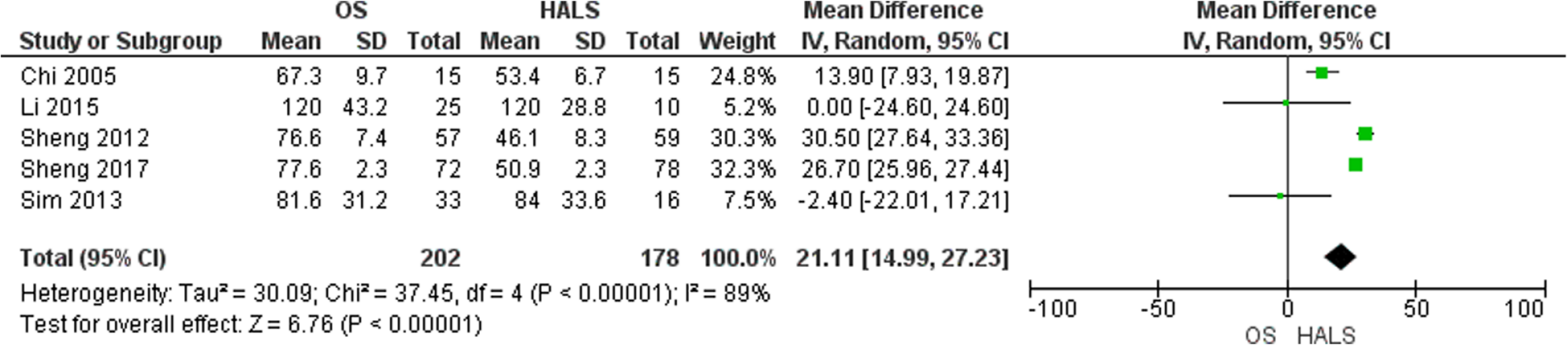
Forest plot of time to first flatus

Five studies [6, 12, 13, 15, 16] mentioned the time of first flatus (Fig. 5). It was reported in 202 patients in the OS group and 178 patients in the HALS group. The time to first flatus was statistically shorter in the HALS group (MD = 21.11, 95% CI 14.99 to 27.23, *p* < 0.00001) with a high heterogeneity level between (*I*^2^ = 89%).

#### Wound infection

**Fig. 6:**
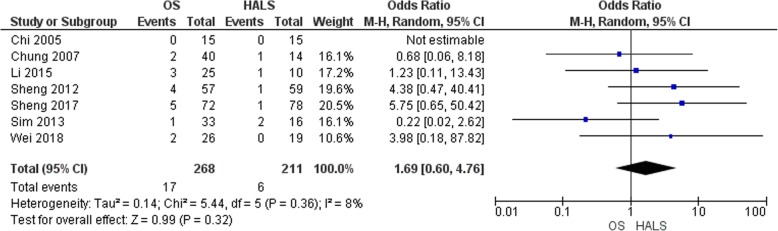
Forest plot of wound infection

All included studies gathered the incidence of wound infection [6, 11–16] (Fig. 6). It was reported in 17 patients out of 268 patients in OS group and 6 patients out of 211 patients in HALS group. There was no evidence of statistical difference (OR = 1.69, 95% CI 0.60 to 4.76, *p* = 0.32) with a low heterogeneity between the studies (*I*^2^ = 8%).

#### Hospital stay

**Fig. 7:**
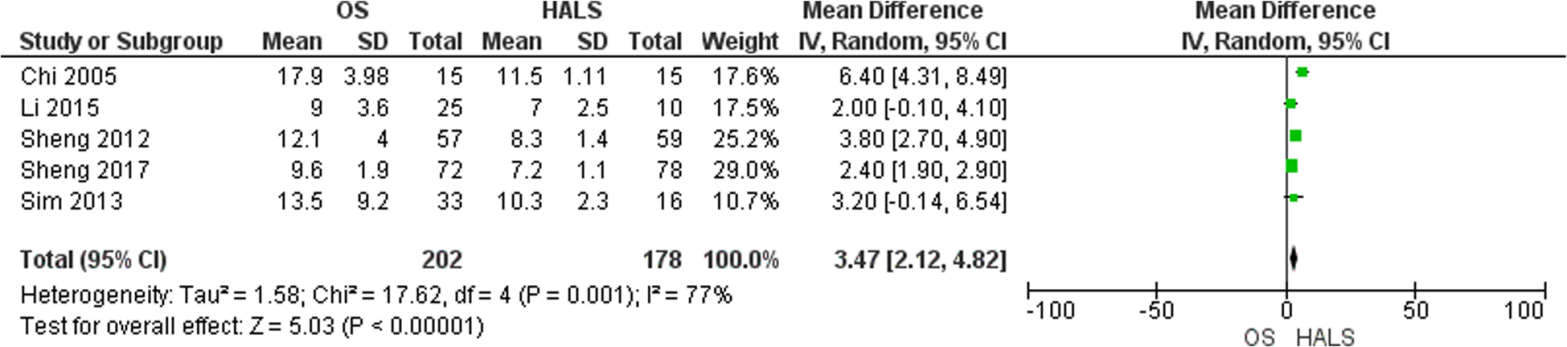
Forest plot of hospital stay

Five studies reported length of hospital stay [6, 12, 13, 15, 16] (Fig. 7). We noticed 202 patients in the OS group and 178 patients in HALS groups. There was a statistically significant shorter hospital stay with HALS than with OS (MD = 3.47, 95% CI 2.12 to 4.82, *p* < 0.00001). There was a little level of heterogeneity, Tau^2^ = 1.58 (*I*^2^ = 77%).

#### Harvested lymph nodes number

**Fig. 8:**
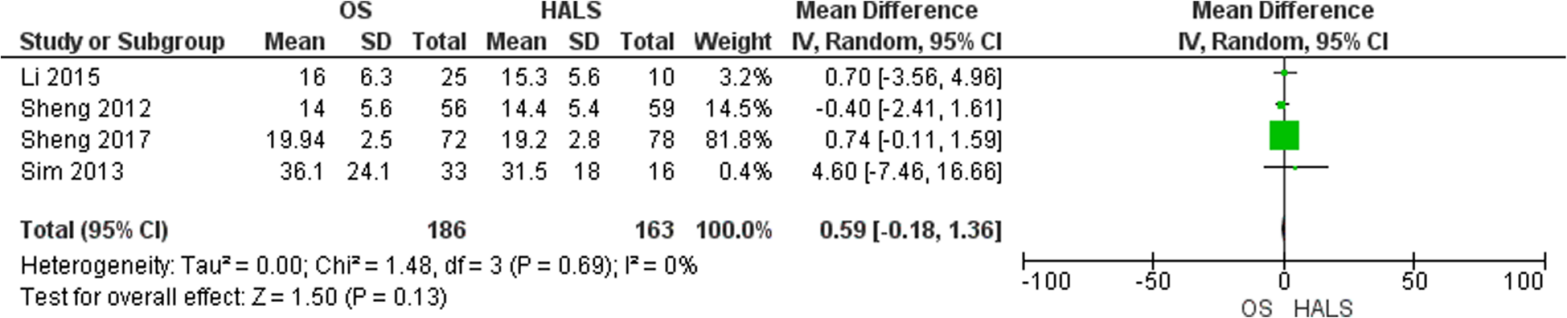
Forest plot of harvested lymph nodes

The number of harvested lymph nodes was presented in four studies [6, 13, 15, 16] (Fig. 8), with 186 patients in the OS group and 163 patients in the HALS group. After pooling the data, no difference was seen (MD = 0.59, 95% CI − 0.18 to 1.36, *p* = 0.13).

#### Postoperative pain score

Three studies [6, 13, 17] reported the operative pain score. Due to heterogeneity between the studies, two studies presented data in the form of means and standard derivation with a lack of standardised protocol for analgesia, and performing a meta-analysis was not appropriate. Less postoperative pain score after HALS than after OS was found.

#### Recurrence

**Fig. 9:**
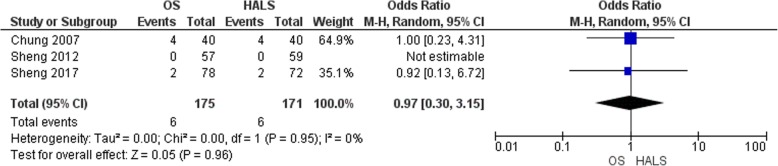
Forest plot of recurrence

Three studies reported the recurrence rate [6, 13, 17] (Fig. 9). This event was reported in six patients out of 175 patients in the OS group and 6 patients out of 171 patients in the HALS groups. There were no differences between these two groups (OR = 0.97, 95% CI 0.30 to 3.15, *p* = 0.96).

### Discussion

This meta-analysis showed that in the group HALS, there were greater outcomes in terms of blood loss, time to first flatus, postoperative pain score, and hospital stay with similar results between these two groups regarding mortality, morbidity, operative time, wound infection, harvested lymph nodes number, and recurrence.

With strong evidence level, laparoscopic approach should be considered standard of care in right colectomy [1, 18]. The anatomical vascular variations, steep learning curve, and lack of long-term oncological outcomes slowed the worldwide spread of standard laparoscopic colectomy. HALS is a stepping stone to conventional laparoscopic surgery. This procedure could be useful for complex cases [7, 19]. However, the vascular pedicle variability of the right colon renders the right hemicolectomy different from the left one [5]. Compared with OS, HALS should warrant the advantages of minimally invasive procedure and at least some oncological-oriented results. In this meta-analysis, we have assessed the results of malignant right colon disease in order to reduce the heterogeneity of colorectal site and type of lesion.

In the seven included studies, a conversion rate was mentioned in three cases by one study. Reasons for conversion were dense adhesions in two cases and right ureteral transection during dissection in one case. Even in case of right hemicolectomy for obstructive right colon cancer or if a CME was performed, no cases of conversion were reported [13, 16]. The conversion rate was 1.26% and ranged from 0 to 7.3%. This rate reflects that HALS is feasible even in complicated cases. In another side, HALS with the added advantages of tactile feedback is correlated to a lower rate of conversion than single-port, laparoscopic-assisted, and totally laparoscopic approaches [5].

One patient in this review died in the hospital. This patient was in the HALS group. He died due to a medical condition: an acute myocardial infarction [17]. The overall mortality rate was of 0.1% in this review, and 0.4% in the HALS group. This demonstrates that both HALS and OS are safe, in case of right colonic cancer, if performed by experienced surgeons. The second important outcome of the HALS is morbidity rate. After pooling the data, the two groups did not differ in terms of overall postoperative complications, essentially the rate of the wound infection.

With regard to the incision length, it was approximately three times shorter in HALS than OS in different studies [6, 13, 16]. This reduced the incision length, significantly decreased abdominal wall complications and postoperative pain, and could affect patient recovery [1, 20].

The results indicate a significant lower blood loss in the HALS group. There was a high heterogeneity rate concerning this criterion. We noticed the absence of standardised method to quantify the blood loss among the studies. Furthermore, Sim et al. [15] evaluated this criteria referring to preoperative and post-operative haemoglobin level. It was 1.6 ± 1.1 g/dl in the OS group and 1.3 ± 0.8 g/dl in the HALS group. This represents one of the most important advantages of HALS that reduces the transfusion-related risks and subsequent morbidity.

These two procedures have similar duration. Some reports mention a longer operative time with mini-invasive right hemicolectomy [3]. In the case of HALS, surgeons introduced their non-dominant hand into the abdomen through a specific hand port to facilitate the procedure and reduce the operative time. Furthermore, an incision is used to retrieve the operative specimen or to perform a digestive anastomosis in the case of conventional laparoscopic or laparoscopic-assisted approach. A high heterogeneity rate between the studies was found in the random effects meta-analysis. This similarity, in terms of operative time, should be considered with cautions. Chi et al. [12] reported a shorter operative time. In addition, if we excluded the study of Chi et al. [12] and the study of Li et al. [16] including cases of acute obstructive right-sided colonic cancer, the heterogeneity decreased to 0% and the operative time became statistically shorter in the OS group.

Time to first flatus and hospital stay display two markers of postoperative recovery. These two criteria were shorter after HALS than OS. However, a high heterogeneity rate was noted between the different studies. This could be due to the absence of standardized postoperative recovery criteria in all these reports. In addition, first liquid diet, first soft diet, and time to ambulation were mentioned in few studies and could be prone to bias because surgeons are likely to start oral intake earlier in HALS than after OS. These results were in congruence with studies comparing mini-invasive surgery to OS [3, 7].

Postoperative pain score was mentioned in three studies [6, 13, 17]. These three studies [6, 13, 17] found less postoperative pain score after HALS than after OS. These results were in harmony with other studies comparing mini-invasive surgery to OS [7].

Total cost has an impact in the widespread application of HALS. For wider adoption, HALS should be at least similar to OS in terms of total cost. In this review, two studies [6, 13] evaluated the total cost. These two studies were performed in China. They showed higher total costs in the HALS group (36200 ± 6993 RMB vs 32544 ± 9774 RMB; *p* = 0.022 and 34660 ± 1458 RMB vs 30721 ± 2135 RMB; *p* = 0.024). This could be due to the hand port and other devices. However, other studies reported also a higher surgical procedure cost but comparable overall cost [21] which reflects fewer complications and expeditious recovery experienced by patients in the HALS [22].

Oncological outcomes after HALS are the centre of recent debates. We assessed in this study the number of harvested lymph nodes and malignant disease recurrence to opt for the best right hemicolectomy approach. These two outcomes were similar in these two right hemicolectomy approaches. However, this cannot be considered as evidence and further RCTs are required to outline the oncological accuracy of HALS role. In addition, incidence of recurrence in case of right colon cancer depends on the type of surgery but also on systematic therapy and tumour stage. We have reported in Table 1 the different tumour stages among the included studies. In the two groups, a systematic therapy was not used before surgery.

In this meta-analysis, several limitations should be considered. We have tried to standardize, but outcome measures were not well-defined. We included two RCTs [6, 17], one prospective non-randomized clinical trial [15], and four CCTs [11–13, 16] in this meta-analysis. This condition could contribute in a selection bias. To overcome this deficiency, the retained studies were rigorously assessed and scored using the methodological index of non-randomized studies (MINORS) and CONSORT statement methods of randomized clinical trials for bias assessment [9, 10]. The professionalism of surgeons and equipment available were important to compare surgical approaches. In our study, six studies were from China and one study was from Korea. Despite the same origins of patients (Asian patients with low BMI), it remains impossible to match all patient groups for tumour grade, stage, and adjuvant chemotherapy, due to the fact that all of these factors can affect oncological outcomes. In addition, the disease-free survival and overall survival rates were not provided in five out of seven studies, and a larger number of patients will be more suitable for oncological safety judgement.

In conclusion, this comprehensive meta-analysis of the available evidence suggests that HALS in right colon cancer is superior to OS in terms of postoperative recovery with similar results in terms of mortality, morbidity, and oncological outcomes. HALS technique should be indicated in the curative management of right-sided colon cancer with a long-term follow-up with oncological outcomes.

## Data Availability

All data generated or analysed during this study are included in this published article [and its supplementary information files].
